# Environmental Factors, Not Biotic Competitive Interactions, Drive the Relative Abundance of Diatoms and Chlorophyta in the Coastal Areas of the Beibu Gulf: Evidence From 18S rDNA Metabarcoding and Partial Least Squares‐Path Modeling Analysis

**DOI:** 10.1002/ece3.71936

**Published:** 2025-08-22

**Authors:** Chunyan Peng, Yuyue Qin, Ying Liu, Dan Sun, Zongsheng Xie, Jixin Jia, Haochen Li, Xiaobo Liu, Hongming Cao, Bin Gong

**Affiliations:** ^1^ The Guangxi Key Laboratory of Beibu Gulf Marine Biodiversity Conservation, College of Marine Sciences Beibu Gulf University Qinzhou China

**Keywords:** Chlorophyta, diatoms, interaction, partial least squares‐path modeling

## Abstract

Diatoms and Chlorophyta are two major phyla of phytoplankton in marine ecosystems. The quantitative detection of the population succession and the interaction between them in natural marine ecosystems is a key challenge that ecologists face. In this study, using high‐throughput sequencing (HTS) analysis, a negative correlation was found between Diatoms and Chlorophyta near the Dafeng River Estuary (DRE) and the Sanniang Bay (SNB) located in the Beibu Gulf, China. To clarify the underlying mechanism, a co‐occurrence network was employed to scrutinize the interspecific relationships between the two phytoplankton groups, and the Mantel test was used to evaluate their relationships with environmental factors. The results indicated that the negative correlation between Diatoms and Chlorophyta was independent of interspecies interactions. Moreover, the effects of environmental factors on Diatoms and Chlorophyta were complex, being both positive and negative across seasons, and thus, they failed to explain this correlation satisfactorily. The partial least squares‐path modeling (PLS‐PM) analysis was performed using six latent variables, including seawater properties, nutrients, biomass, alpha diversity, Chlorophyta, and Diatoms. According to the results, the mechanisms behind the negative correlation between Chlorophyta and Diatoms varied across different seasons. Overall, both the differing responses of Chlorophyta and Diatoms to changes in temperature and nutrients and the complex hydrodynamic characteristics of the estuary and the bay in the study area were the main factors causing this negative correlation. This study offers a new approach to understand the succession of some phyla in phytoplankton.

## Graphical abstract

Using high‐throughput sequencing (HTS) analysis, a negative correlation was found between Bacillariophyta and Chlorophyta near the Dafeng River Estuary (DRE) and the Sanniang Bay (SNB) located in the Beibu Gulf, China. The partial least squares‐path modeling (PLS‐PM) analysis showed that both the differing responses of Chlorophyta and Bacillariophyta to changes in temperature and nutrients and the complex hydrodynamic characteristics of the estuary and the bay were the main factors causing this negative correlation.

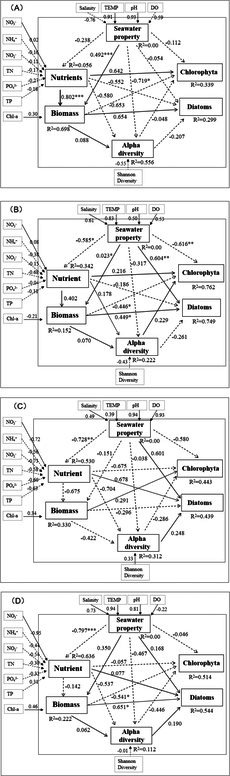

## 1 Introduction

Diatoms represent one of the most biodiverse taxonomic groups on Earth (Nakov et al. 2018). They make significant contributions to the abundance of species that are found in both marine and freshwater ecosystems (Clement et al. 2017). Diatoms play critical roles in the process of the biological carbon pump by assimilating organic carbon in the oceanic systems (Tréguer et al. 2018). Chlorophyta is a taxonomic group of algae in aquatic environments with the smallest size classes, including pico‐plankton (0.2–2 μm) and nano‐plankton (2–20 μm) (Lin et al. 2021). Chlorophyll b, which acts as the main accessory pigment, is present in Chlorophyta (Lopes‐dos‐Santos et al. 2016). Several classes, including Chloropicophyceae, Mamiellophyceae, Nephroselmidophyceae, Palmophyllophyceae, Pedinophyceae, Picocystophyceae, Pyramimonadophyceae, and Ulvophyceae, belong to the phylum Chlorophyta. Except for a rather small proportion of these classes, e.g., Pedinomonadales in Pedinophyceae, they mostly have been discovered in the oceanic environment (Tragin et al. 2016).

Diverse species of phytoplankton occupy marine habitats. There is a critical link between the alterations in phytoplankton communities and fluctuations in ecosystem health (Zhou, Dong, et al. 2024; Zhou, Liu, and Li 2024; Klisarova et al. 2023). A significant challenge is to understand the assembly mechanisms of marine phytoplankton (Zhao et al. 2021; Rojo 2021). Diatoms and Chlorophyta are two important groups of plankton in marine ecosystems. The mechanisms of community assembly and the interspecific interactions between them are of considerable importance (Hou et al. 2020). A large body of research has simulated the interactions among more than two phytoplankton taxa in the laboratory (Yan et al. 2020). However, the research on the interspecific relationships between Diatoms and Chlorophyta in natural marine environments is scarce. An efficient technique for quantifying phytoplankton biodiversity is high‐throughput sequencing (HTS) (Liu et al. 2021; Zhang et al. 2020). Ecologists have derived immense value from the burgeoning research on phytoplankton communities by metabarcoding (Liu, Wang, et al. 2020; Liu, Gibson, et al. 2020). The examination of the phytoplankton interspecific relationships in natural ecosystems is entirely possible by analyzing the HTS datasets (Boyse et al. 2024).

The Beibu Gulf, located in the northwestern South China Sea (SCS), is an important region for aquaculture production (Su et al. 2022). Sanniang Bay (SNB) and Dafeng River (DFR) in the northern Beibu Gulf are considered habitats for Indo‐Pacific humpback dolphins ( *Sousa chinensis* ) (Peng et al. 2020). However, the Beibu Gulf is a shallow, semi‐enclosed gulf (Su et al. 2022), where eutrophication mostly occurs due to the continuous use of agricultural fertilizers, industrial advances, and anthropological activities (Li et al. 2020; Lao et al. 2021). However, the questions that arise under such conditions are as follows: (1) how the interaction between Diatoms and Chlorophyta is established at temporal and spatial scales? and (2) is this interaction influenced more by the environmental factors or the species interactions?

In this study, a negative correlation was found between the abundance of Diatoms and Chlorophyta. The mechanism underlying this correlation was elucidated by employing the co‐occurrence network analysis to explore the interspecific relationships between them and using the Mantel test to examine the relationships between environmental variables and phytoplankton groups, which, however, failed to offer a satisfactory explanation. The partial least squares‐path modeling (PLS‐PM) method, also called partial least squares structural equation modeling (SEM), has a wide application for evaluating complex models specifying the interactions between latent variables (Rodrigues et al. 2019). More frequent use of PLS‐PM in the social sciences has been reported (Crocetta et al. 2021). Moreover, there is a growing literature analyzing the cause‐effect relationships between anthropogenic pressures and their ecological impacts (Fernandes et al. 2019). We hypothesized that the factors exerting influences on phytoplankton can be divided into two categories. The first category included seawater characteristics, consisting mainly of physical properties such as temperature, dissolved oxygen, pH, and salinity, as well as nutrients. The abundance represented by Chl‐a concentration and diversity, e.g., alpha diversity, of phytoplankton, which were affected by the factors from the first category, made up the second category. The factors in the first category could induce direct alterations in the abundance of Diatoms and Chlorophyta. The effects of the physical characteristics of seawater and nutrients on the abundance of Diatoms and Chlorophyta could be more clearly seen with the combination of the first and second categories. In this paper, PLS‐PM analysis was performed using six latent variables, including seawater properties, nutrients, biomass, alpha diversity, Chlorophyta, and Diatoms, to unravel the mechanism of the negative correlation between these two phytoplankton classes. Therefore, this research can provide a new way to understand the interactions between them in aquatic ecosystems.

## 2 Materials and Methods

### 2.1 Field Sampling and Measurement of Environmental Factors

**FIGURE 1:**
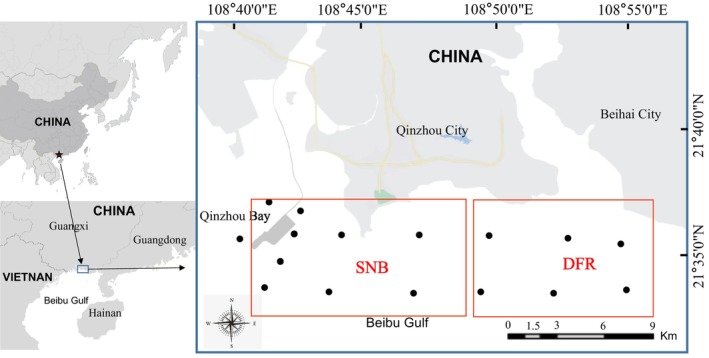
Sampling sites in the Dafeng River (DFR) estuary and the Sanniang Bay (SNB) in the northern Beibu Gulf, China. Black dots denote sampling sites, red frames delineate the partitioning of these sampling locations into two zones (DRE and SNB), and gray areas represent terrestrial regions.

A total of 16 sites with a salinity of 17.78‰–31.99‰ and a depth of less than 11 m were selected for sampling, which are the same as those used in our previous study (Figure 1) (Xiong et al. 2024). Samples were collected from the Dafeng River estuary (DRE) and the Sanniang Bay (SNB) located in the northern Beibu Gulf, China (21.63° N, 108.69° E—21.56° N, 108.93° E) in March, July, September, and December from 2018 to 2019. The procedures for the collection and treatment of all seawater samples were the same as the published article (Xiong et al. 2024).

The environmental factors were evaluated using the methods described in our published article (Xiong et al. 2024). These environmental factors included dissolved oxygen (DO), ammonium (NH_4_^+^), nitrite (NO_2_^−^), nitrate (NO_3_^−^), phosphate (PO_4_^3−^), total organic carbon (TOC), total nitrogen (TDN), chlorophyll a (Chl‐a), temperature (TEMP), depth, salinity, and pH. A portable electrical equipment was used for the on‐site measurement of TEMP, depth, salinity, pH, and DO. NH_4_^+^, NO_2_^−^, NO_3_^−^, PO_4_^3−^, TOC, TDN, and Chl‐a concentrations were determined after the transport of 2 L of seawater to the laboratory, followed by filtering with 0.45‐μm nylon filters (Xiong et al. 2024). The equipment specifications can be obtained from the published article (Xiong et al. 2024).

### 2.2 DNA Extraction and PCR Amplification and Sequencing

The environmental DNA (eDNA) was extracted from seawater samples by filtering 500 mL of seawater through 0.45‐μm PVDF membranes. The remaining membrane was used to extract DNA using the Water Filter DNA Extraction Kit (GBCBIO Technologies Inc., China). After the quantification of the obtained DNA from the samples from each sampling site using NanoDrop One (Thermo Fisher Scientific, USA), it was used for the amplification of the V4 subregion of the 18S rDNA gene (Tytgat et al. 2019). High‐throughput sequencing (HTS) was performed on the HiSeq2500 sequencing platform in Shanghai Personal Biotechnology Co. Ltd., China. Following the acquisition of raw data, data processing was carried out in several sequential steps. The merging of paired‐end reads was performed with PEAR software (v0.9.6) (Zhang et al. 2014) after the removal of adapters and low‐quality regions using SeqPurge v0.1–852‐g5a7f2d2 (Sturm et al. 2016), followed by removing the chimeric and barcode sequences using UCHIME method with default parameters (Edgar et al. 2011). For each pipeline, the standard commands were utilized. Sequences with more than 97% similarity to the representative sequences were clusterd into OTUs using the Usearch (V7.0) (Zhou, Dong, et al. 2024; Zhou, Liu, et al. 2024; He et al. 2023). Thereafter, the taxonomy for each OTU was annotated using the SILVA SSU r138.1 taxonomy information provided by mothur (Perry et al. 2024). Algal OTUs were taxonomically verified using AlgaeBase (Guiry and Guiry 2019). The abundance of algae was calculated as the number of sequences assigned to a given taxon (Tables S1–S4) (Liu, Wang, et al. 2020; Liu, Gibson, et al. 2020). The relative abundance table of phyla Chlorophyta, Diatoms, Cryptophyceae, and Streptophyta were constructed using a custom R script (available in Code S1).

### 2.3 Statistical Analysis

Pearson correlation was employed to examine the relationships between the abundance of Chlorophyta and Diatoms. Only those interactions among OTUs of phytoplankton groups that were statistically significant (*p* < 0.05) and significantly strong (*r* > 0.8) were used to construct the correlation network (Lin et al. 2019) using the “Hmisc” package in R software (Harrell‐Jr and Harrell‐Jr 2019). The Gephi software version v0.9.2 was used to visualize the networks (Bastian et al. 2009). To analyze the relationships between the environmental factors and the abundance of phytoplankton (Table S5–S12), the Mantel test was performed using the “reshape2” package in R (Meng et al. 2022), and the R code is available in Code S2.

Partial least squares‐path modeling (PLS‐PM) analysis (R code is available in Code S3) was performed using the “plspm” package in R (Sanchez et al. 2013) to explain the formation mechanism of the countervailing trends for Chlorophyta and Diatoms. The selected latent variables, e.g., seawater properties, nutrients, phytoplankton biomass represented by the Chl‐a concentration, alpha diversity, Chlorophyta, and Diatoms, were used to construct the path model (Table S13–S16). The PLS‐PM path model consists of two models, namely the inner model (structural model) and the outer model (measurement model) (Fernandes et al. 2018). The inner model signifies the relationships among the latent variables (Henseler and Chin 2010). Seawater characteristics, particularly temperature and salinity, directly influenced not only the phytoplankton abundance but also the distribution of nutrients. For example, the significant increase in nutrient concentrations by runoff in the Dafeng River estuary (DRE) during the rainy season (July and September) was found to be highly associated with the salinity of seawater. The nutrients were not directly affected by seawater properties; instead, they might undergo conversion from the organic matter released from the lysis of algal cells. Seawater properties and nutrients caused alterations in the abundance (Chl‐a) and diversity of phytoplankton, which reflect the competition or synergy within the community, significantly affecting both Chlorophyta and Diatoms. The indicators of latent variables and their correlations are mainly described by the outer model.

## 3 Results

### 3.1 The Abundance of Diatoms and Chlorophyta in the Sampling Regions

**FIGURE 2:**
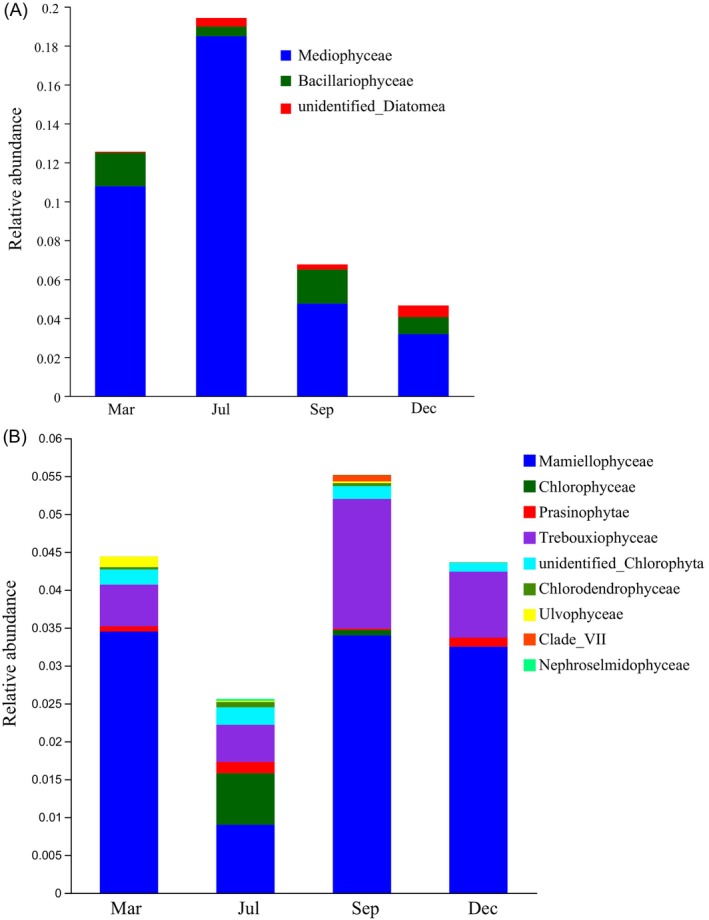
Relative abundance of Diatoms and Chlorophyta in sampling sites in the four seasons. (A) The relative abundance of Diatoms. (B) The relative abundance of Chlorophyta. The vertical axis denotes the relative abundance of the dominant classes of Diatoms or Chlorophyta, whereas the horizontal axis represents the four seasons (Mar: March; Jul: July; Sep: September; Dec: December).

Figure 2 shows the relative abundance of Diatoms and Chlorophyta. The predominant Diatom classes were Mediophyceae, Bacillariophyceae, and unidentified Diatomea, while Mamiellophyceae, Chlorophyceae, Prasinophytae, Trebouxiophyceae, unidentified Chlorophyta, Chlorodendrophyceae, Ulvophyceae, Clade VII, and Nephroselmidophyceae were the most abundant classes of Chlorophyta, with the higher relative abundance of Diatoms than that of Chlorophyta, particularly in March and July.

### 3.2 The Negative Correlation Between Diatoms and Chlorophyta

**FIGURE 3:**
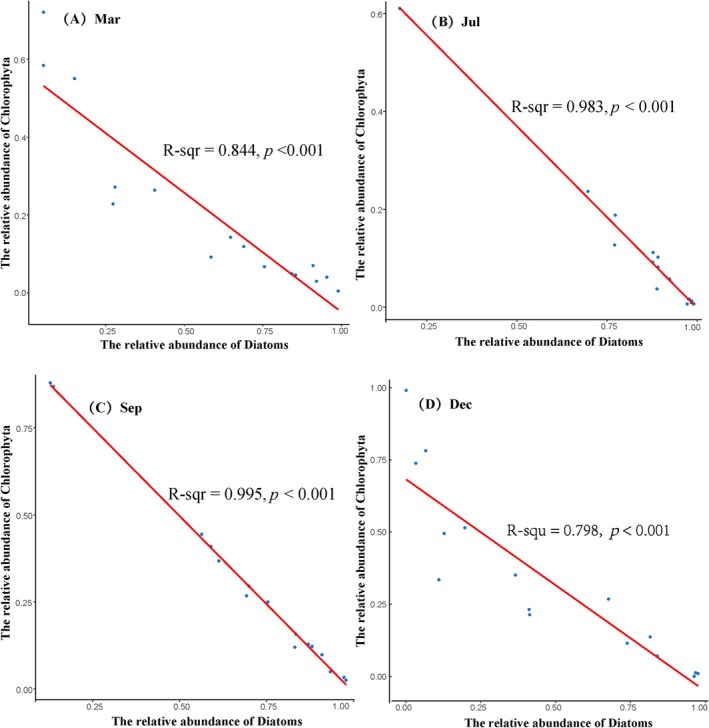
Pearson's correlation showing a statistically significant negative correlation between relative abundance of Diatoms and Chlorophyta in the sampling sites (A, B, C, and D indicate the correlation between Diatoms and Chlorophyta in March, July, September and December, respectively). The horizontal axis shows the relative abundance of Diatoms, and the vertical axis shows the relative abundance of Chlorophyta.

The 18S rDNA metabarcoding data revealed the initial increase in the abundance of Diatoms in March and July, reaching its peak in the latter, followed by a decrease in September and December, with the lowest value obtained in the latter. However, the abundance of Chlorophyta first declined in March and July, with the latter exhibiting the minimum value, and then rose in September and December, with the peak occurring in the former. The abundances of both Diatoms and Chlorophyta across the four seasons, however, followed an opposite changing trend. A statistically significant negative correlation between the abundance of these two phytoplankton groups was noted in March (*R* ^2^ = 0.844, *p* < 0.001), July (*R* ^2^ = 0.983, *p* < 0.001), September (*R* ^2^ = 0.995, *p* < 0.001), and December (*R* ^2^ = 0.798, *p* < 0.001), as revealed by Pearson correlation analysis (Figure 3).

### 3.3 The Co‐Occurrence Network Analysis Reveals the Interaction Between Diatoms and Chlorophyta

To evaluate whether the observed negative correlation between Diatoms and Chlorophyta resulted from the direct interspecific interactions, a comprehensive co‐occurrence network analysis was performed. Four seasons were not significantly different in terms of the number of OTUs (nodes) in co‐occurrence networks. Nevertheless, the number of links among nodes in March (326) and July (421) surpassed those in September (187) and December (224), which implies a far more intricate network of interactions in March and July than in September and December.

**FIGURE 4:**
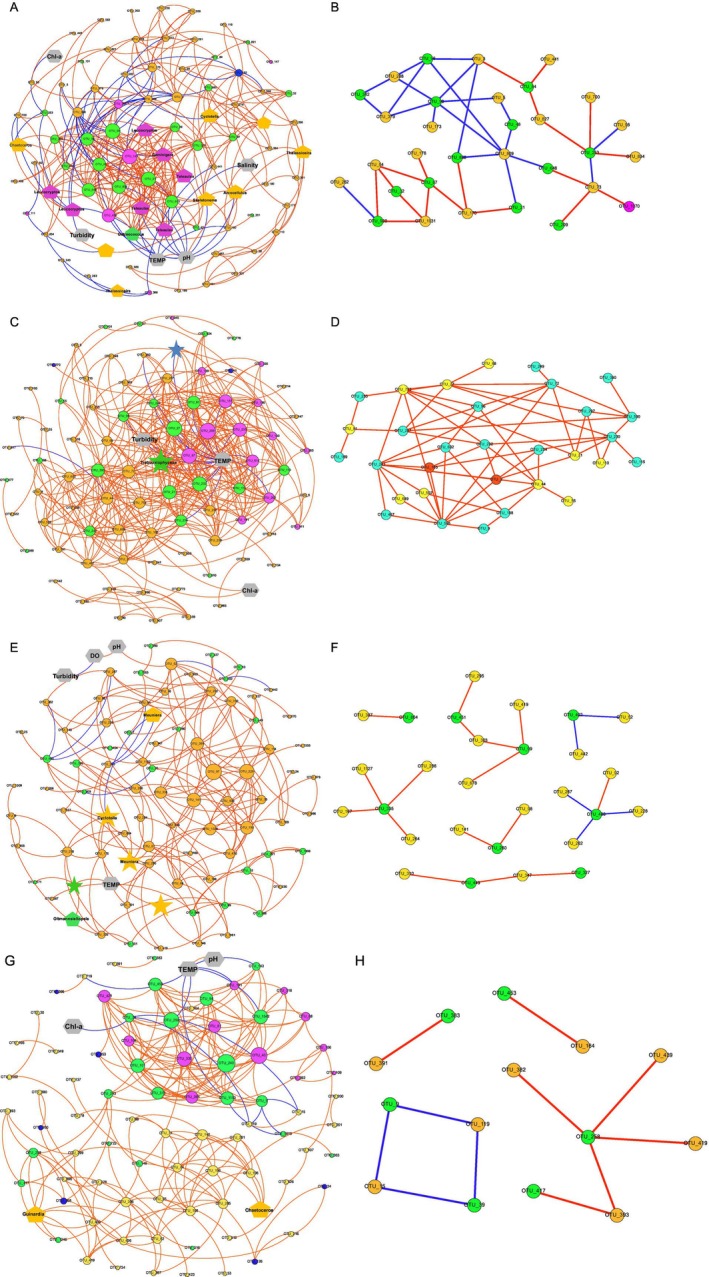
Co‐occurrence network analysis showing the interactions among phytoplankton OTUs and environmental factors. A, C, E, and G indicate the co‐occurrence networks for all of the phytoplankton OTUs in March, July, September, and December, respectively. B, D, F, and H indicate the interaction networks among Diatoms and Chlorophyta in March, July, September, and December, respectively. The red or blue lines in the networks from panels A‐H indicate positive or negative interactions between OTUs. In panels A, C, E, and G, the interactions among environmental factors (represented by 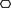) and OTUs were included in the network. The OTUs with significantly higher abundance in SNB are represented by 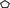, whereas those with significantly higher abundance in DFR are denoted as 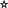.

The main taxa in co‐occurrence networks in March were Diatoms (49 nodes), Chlorophyta (21 nodes), and Cryptophyceae (13 nodes). Some OTUs belonging to Diatoms, Chlorophyta, and Cryptophyceae formed the largest module in the network. Several OTUs, including 7 in Cryptophyceae and 8 in Diatoms, were highly enriched in samples from the SNB region. The environmental factors, especially pH and temperature, were more intricately connected to OTUs (Figure 4A). Moreover, the direct positive and negative interactions between Diatoms and Chlorophyta in the network were demonstrated (Figure 4B).

The dominant groups in July were Diatoms (45 nodes), Chlorophyta (21 nodes), and Cryptophyceae (14 nodes). There were two primary modules in the network, with one predominantly consisting of Diatoms, while Chlorophyta and Cryptophyceae were the groups that mainly constituted the other. A stronger linkage was observed among turbidity, temperature, and OTUs (Figure 4C). Diatoms and Chlorophyta interacted only directly and positively in the network (Figure 4D).

In September, the co‐occurrence network was dominated by the taxa, including Diatoms (61 nodes) and Chlorophyta (23 nodes). Two Diatoms OTUs and 1 Chlorophyta OTU were significantly enriched in DFR, but only 1 OTU had a greater abundance in SNB. More linkages were noticed between temperature and OTUs (Figure 4E). Diatoms and Chlorophyta established both positive and negative interactions in the network (Figure 4F).

In December, Diatoms (43 nodes), Chlorophyta (21 nodes), and Cryptophyceae (12 nodes) dominated the network. The network consisted of two primary modules, with the first one mainly comprising Chlorophyta and Cryptophyceae, while in the other, Diatoms were predominant. A larger number of linkages was found among environmental variables, e.g., temperature and pH, and phytoplankton groups (Figure 4G). As seen in Figure 4H, there were both positive and negative interactions between Diatoms and Chlorophyta in the network.

### 3.4 The Interactions Between Environmental Factors and Diatoms and Chlorophyta

**FIGURE 5:**
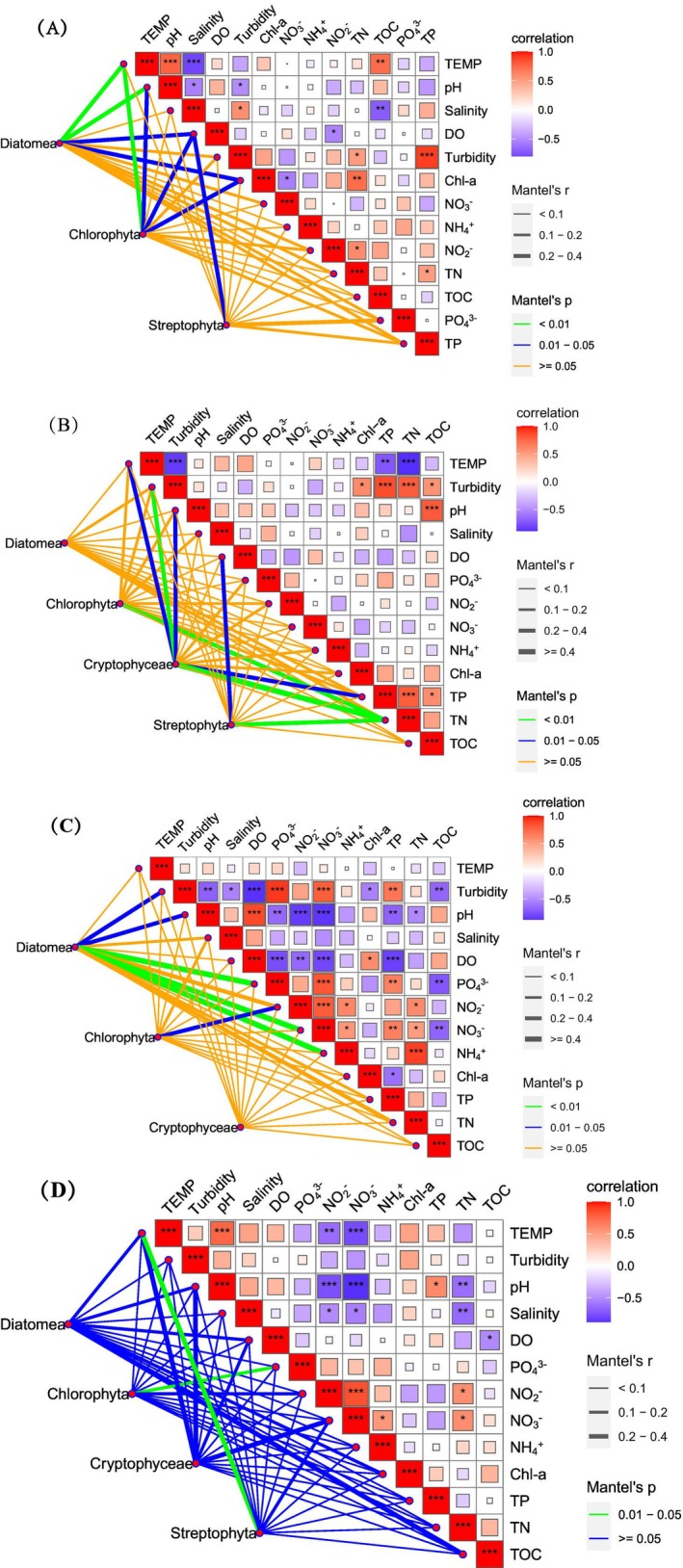
Mantel's test revealing the relationships between the environmental factors and the abundance of phytoplankton groups, including Chlorophyta, Diatoms, Cryptophyceae, and Streptophyta (A, B, C, and D indicate the relationships between the environmental factors and the phytoplankton in March, July, September, and December, respectively). The boxes denote the magnitudes of Pearson's correlation coefficients between environmental factors, where red indicates a positive correlation (R > 0), with darker shades of red representing higher correlation coefficients; blue indicates a negative correlation (R < 0), with darker shades of blue corresponding to higher correlation coefficients. The correlations between environmental factors and phytoplankton abundance were calculated using the Mantel test. The magnitude of the correlations is represented by line segments: The higher the correlation coefficient (Mantel's r), the thicker the line segment. The significance of the correlations (Mantel's p) is denoted by line segments of different colors.

Environmental factors were found to establish correlations, which were the most pronounced in September. Multiple strong positive correlations were observed, which were between turbidity and TP, turbidity and NO_3_^−^, turbidity and PO_4_^3−^, pH and DO, NO_3_^−^ and PO_4_^3−^, TP and PO_4_^3−^, NO_3_^−^ and NO_2_^−^, TP and NO_3_^−^, and TN and NH_4_^+^. In contrast, turbidity and pH, turbidity and DO, turbidity and TOC, pH and PO_4_^3−^, pH and NO_2_^−^, pH and NO_3_^−^, pH and TP, DO and PO_4_^3−^, and DO and NO_3_^−^ exhibited negative correlations (Figure 5).

The relationship between Diatoms/Chlorophyta and environmental factors demonstrated significant variations in July, September, and December. Moreover, Chlorophyta was significantly related to TN (*p* < 0.01) in July and to PO_4_^3−^ (*p* < 0.01) in December. In September, Diatoms displayed significant correlations with turbidity (*p* < 0.05), pH (*p* < 0.05), PO_4_^3−^ (*p* < 0.01), NO_3_^−^ (*p* < 0.01), and NH_4_^+^ (*p* < 0.01), while Chlorophyta established a significant correlation with only NO_2_^−^ (*p* < 0.05) (Figure 5). In March, however, significant relationships existed between both Diatoms and Chlorophyta and some environmental variables, including temperature (*p* < 0.01), pH (*p* < 0.01 for the former and *p* < 0.05 for the latter), DO (*p* < 0.05), and Chl‐a (*p* < 0.05). Therefore, environmental factors could not fully explain the negative correlation between these two phytoplankton groups.

### 3.5 The Negative Correlation Between Diatoms and Chlorophyta Revealed by PLS‐PM Analysis

**FIGURE 6:**
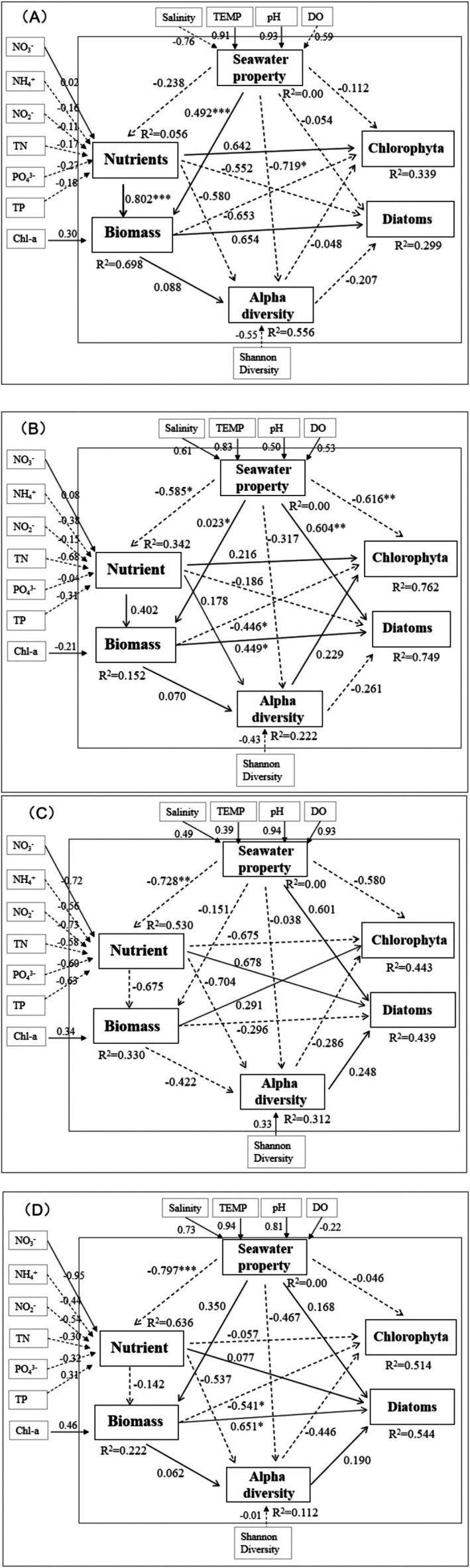
Partial least‐squares path modeling (PLS‐PM) showing the impacts of latent variables (seawater properties, nutrients, phytoplankton biomass represented by the Chl‐a concentration, and alpha diversity) on Chlorophyta and Diatoms. A, B, C, and D indicate the cause–effect relationships between the environmental factors and the phytoplankton in March, July, September, and December, respectively. Dotted lines represent negative relationships, whereas solid lines indicate positive relationships; ***p < 0.001, **p < 0.01, and *p < 0.05. The PLS‐PM path model consists of two models, namely, the inner model (including latent variables in the area enclosed by the inner large square frame) and the outer model (including latent variables in the area between the outer large square frame and the inner large square frame).

To evaluate the relationships among the latent variables (seawater properties, nutrients, phytoplankton biomass, alpha diversity, and Diatoms/Chlorophyta), the PLS‐PM analysis was performed (Figure 6). In March, the nutrients and seawater properties contributed to the enhancement of biomass (*R* = 0.492, *p* < 0.001), which was further positively correlated with Diatoms (*R* = 0.654) but negatively correlated with Chlorophyta (*R* = −0.653). Moreover, there was a positive correlation between nutrients and Chlorophyta (*R* = 0.642), whereas the former established a negative correlation with Diatoms (*R* = −0.552). In July, two lines were related to Chlorophyta and Diatoms. The first one showed the direct impact of seawater properties on the abundance of Chlorophyta (*R* = −0.616, *p* < 0.01) and Diatoms (*R* = 0.604, *p* < 0.01). The second demonstrated the impact on nutrients exerted by seawater properties (*R* = −0.585, *p* < 0.05), affecting biomass (*R* = 0.402), which ultimately led to a positive effect on Diatoms (*R* = 0.449, *p* < 0.05) but a negative effect on Chlorophyta (*R* = 0.446, *p* < 0.05). In September, two lines exhibited correlations with Chlorophyta and Diatoms, including the effects of seawater properties on the density of Chlorophyta (*R* = −0.580) and Diatoms (*R* = 0.601) and on nutrients (*R* = −0.728, *p* < 0.01), further influencing Diatoms (*R* = 0.678) and Chlorophyta (*R* = −0.675). In December, however, only one line was associated with Chlorophyta and Diatoms, that is, the seawater properties influencing biomass (*R* = 0.350), which subsequently affected both Diatoms (*R* = 0.651, *p* < 0.05) and Chlorophyta (*R* = −0.541, *p* < 0.05).

## 4 Discussion

Aquatic organisms make up a complex ecosystem that is comprised of interacting phytoplankton communities as its crucial components (Jackrel et al. 2021). The positive or negative interactions among phytoplankton groups revealed by Pearson correlation analysis were mostly competitive or mutualistic (Roy et al. 2007). High‐throughput sequencing used in the present study showed a negative correlation between Diatoms and Chlorophyta in marine habitats. The competitive interaction between Diatoms and Chlorophyta was discovered in previous research. For example, the possibility of interspecific competition among three selected typical species of Cyanobacteria, Chlorophyta, and Diatoms, including *Microcystis aeruginosa* , *Scenedesmus quadricauda* , and *Cyclotella* sp., respectively, in response to nitrate pulse supply was examined (Wen et al. 2012). However, the competitive relationship between Diatoms and Chlorophyta at the phylum level has not been reported previously, which, given the intricacy of marine ecosystems, may be of greater importance.

The interspecific interactions among organisms in aquatic ecosystems were both complex and difficult to quantify (Røder et al. 2020). Nevertheless, ecologists can infer the microeukaryote—bacteria, bacteria—bacteria, and microeukaryote—microeukaryote interactions from both HTS datasets and statistical analysis of their spatial co‐occurrence patterns (Zheng et al. 2023; Yang et al. 2020; Thurman et al. 2019). The co‐occurrence network analysis was performed to ascertain whether the negative correlation between Diatoms and Chlorophyta was caused by the interactions among species within these two phytoplankton groups. Crucially, the co‐occurrence network analysis revealed predominantly positive or mixed interspecific interactions between Diatoms and Chlorophyta OTUs. This disconnects the abundance pattern from direct competitive exclusion.

The varying and significant influences of physicochemical parameters of seawater on phytoplankton were reported (Vajravelu et al. 2018). Diatoms are one of the major primary producers in marine ecosystems (Glud et al. 2002). They are highly sensitive to changes occurring in aquatic environments, such as eutrophication and pollution (Shibabaw et al. 2021; Kim et al. 2019; Sommer 1994; Colijn and Van‐Buurt 1975). Chlorophyta are green algae found in coastal regions where long‐term eutrophication occurs (Salo and Salovius‐Laurén 2022). Moreover, temperature was identified as a key factor facilitating excessive growth and colonization of Chlorophyta in spring and summer (Deng et al. 2012). However, the competitive relationship between Diatoms and Chlorophyta, which is contributed by seawater physicochemical parameters in the coastal zone, has not been thoroughly examined recently. To elucidate whether the competition between Diatoms and Chlorophyta is induced by environmental conditions, the Mantel test was employed. Environmental factors divergently affected both Diatoms and Chlorophyta in July, September, and December. However, the Mantel test failed to identify consistent shared environmental pressures that could directly force the inverse abundance relationship.

The PLS‐PM analysis resolved this paradox by uncovering indirect causal pathways linking environmental factors to the negative correlation. Key mechanisms include:

### 4.1 Hydrodynamically Modulated Nutrient Pathways (Rainy Season: July and September)

Increased terrestrial discharge altered seawater properties (salinity, DO) and nutrient regimes during the rainy season (July & September). Hydrodynamic complexity (e.g., stratification in SNB vs. mixing in DFR) created spatially heterogeneous environments. Inconsistent nutrient mobility and DO levels between sites (e.g., low DO in stratified SNB vs. higher DO in mixed DFR) differentially favored Diatoms and Chlorophyta (Bai and Huang 2025). Moreover, in the DFR, the concentrations of nitrate (NO_3_^−^) and ammonium (NH_4_^+^) were relatively higher, whereas in the SNB, the concentration of nitrite (NO_2_^−^) may be relatively higher due to anaerobic conditions. Crucially, Diatoms and Chlorophyta exhibited divergent nutrient preferences (Andersen et al. 2020). The Mantel test also showed that, in September, Diatoms displayed significant correlations with NO_3_^−^ and NH_4_^+^, while Chlorophyta established a significant correlation with NO_2_^−^. These hydrodynamically driven nutrient shifts directly opposed the groups' success.

### 4.2 The Impact of Nutrients and Seawater Properties (Especially, the Temperature) on Biomass and its Implications

PLS‐PM analysis showed that, in March, nutrients and seawater properties facilitated biomass augmentation, which itself was found to be positively correlated with Diatoms but negatively correlated with Chlorophyta. Moreover, a relatively little contribution of inorganic nutrients, such as NO_3_^−^, NH_4_^+^, NO_2_^−^, and PO_4_^3−^, was found to be correlated with nutrients. Nutrients were not only necessarily inorganic nitrogen supplied by terrigenous inputs but also by converting dissolved organic matter (DOM) released from algal cell cytolysis, such as the lysis of *Phaeocystis globosa* in March (Rauch et al. 2008; Grossart and Ploug 2001). This is associated with the phenomenon that seawater properties were not correlated with nutrients, which, however, had a strong correlation with biomass, as shown by PLS‐PM analysis. The Mantel test also showed that in March, Diatoms and Chlorophyta displayed significant correlations with pH, DO, and Chl‐a, changes in which frequently relate to the lysis of algae. The silicon and organic nitrogen released after algae lysis in seawater help Diatoms survive, leading to the development of competitive advantages, and ultimately restricting the growth of Chlorophyta (Wilhelm et al. 2006). Moreover, large quantities of algae cause seawater acidification by decreasing pH and close links between seawater properties and biomass.

PLS‐PM analysis showed that significant impacts of seawater properties were positive on biomass but negative on the diversity of phytoplankton in March and December, which were more apparent in the former. We hypothesize that the increase in values of seawater properties, especially the temperature rise, caused an increase in only the abundance of dominant cold‐adapted species but reduced diversity. Moreover, biomass was positively related to Diatoms but established a negative correlation with Chlorophyta. This implies that seawater properties (most likely, the temperature) may have positively contributed to the dominance of cold‐adapted Diatom species. Coincidentally, there have been reports of an increase in algal biomass and the selective growth promotion of Diatoms by temperature rise in March (Zhang et al. 2019; Bi et al. 2021).

### 4.3 Seasonally Variable Nutrient‐Diversity‐Biomass Interactions

The impact of nutrients and seawater properties on community structure (biomass and diversity) varied seasonally, reinforcing the negative correlation. In March, nutrients/seawater properties boosted biomass but reduced diversity, favoring cold‐adapted Diatoms dominance over Chlorophyta. In July, contrastingly, nutrients enhanced both biomass and diversity, promoting the growth of all algae, which is consistent with the general situation (Chang et al. 2022). However, contrary to common knowledge, in September, the negative correlation between nutrients and biomass was evident. Elevated nutrient levels in the DFR may result from terrestrial inputs, where vertical water mixing occurs and seawater temperatures exceed the optimal range for phytoplankton growth. In contrast, SNB stratification creates a cooler subsurface layer favorable for phytoplankton growth, thereby increasing biomass. In December, low temperatures combined with nutrient inputs favored only cold‐adapted specialists (predominantly Diatoms), decreasing overall diversity while maintaining the inverse biomass relationship with Chlorophyta.

Our study challenges the assumption that negative abundance correlations at high taxonomic levels (phylum) necessarily reflect direct competition. By combining metabarcoding with PLS‐PM, we provide a framework to disentangle environmental mediation from biotic interactions in complex plankton communities. The hydrodynamic complexity of estuaries and bays like the Beibu Gulf amplifies these indirect effects, making PLS‐PM particularly valuable for such dynamic systems. This mechanistic understanding is crucial for predicting phytoplankton succession under changing nutrient loads and climate regimes.

## 5 Conclusions

1. The competitive relationship between Diatoms and Chlorophyta at the phylum level was shown by the analysis of high‐throughput sequencing (HTS).
2. The co‐occurrence network analysis revealed the positive interactions among Diatoms and Chlorophyta species in July. Moreover, during other seasons, there were both positive or negative inter‐relationships between Diatoms and Chlorophyta, indicating that the interspecies interactions could not explain the negative correlation observed between the two plankton groups.
3. The Mantel test showed the complex influence of environmental factors on Diatoms and Chlorophyta across seasons. For example, the strikingly similar effects of environmental variables on both plankton classes were observed in March. However, environmental factors alone could not explain the negative correlation found between them.
4. According to the PLS‐PM analysis, different responses of Chlorophyta and Diatoms to temperature and nutrient changes, as well as the complex hydrodynamic characteristics of the estuary and bay tested in this study, mainly contributed to the negative correlation between the two.

## Author Contributions

**Chunyan Peng:** data curation (equal), software (equal), writing – original draft (equal). **Ying Liu:** data curation (equal), software (equal), writing – original draft (equal). **Yuyue Qin:** data curation (equal), software (equal), writing – original draft (equal). **Dan Sun:** data curation (equal), methodology (equal), writing – review and editing (equal). **Jixin Jia:** data curation (equal), methodology (equal), writing – review and editing (equal). **Zongsheng Xie:** data curation (equal), methodology (equal), writing – review and editing (equal). **Haochen Li:** data curation (equal), methodology (equal), writing – review and editing (equal). **Xiaobo Liu:** data curation (equal), methodology (equal), writing – review and editing (equal). **Hongming Cao:** data curation (equal), methodology (equal), writing – review and editing (equal). **Bin Gong:** conceptualization (equal), funding acquisition (equal), investigation (equal), methodology (equal), supervision (equal), validation (equal).

## Conflicts of Interest

The authors declare no conflicts of interest.

## Supporting information


**Appendix S1:** ece371936‐sup‐0001‐AppendixS1.docx.

**Appendix S2:** ece371936‐sup‐0002‐Supinfo.zip.

## Data Availability

Raw sequence reads are deposited in the SRA (Bioproject PRJNA1060426).
