# Supplementary material for: Environmental Factors, Not Biotic Competitive Interactions, Drive the Relative Abundance of Diatoms and Chlorophyta in the Coastal Areas of the Beibu Gulf: Evidence From 18S rDNA Metabarcoding and Partial Least Squares‐Path Modeling Analysis
Source: Ecol Evol. 2025 Aug 22;15(8):e71936. doi: 10.1002/ece3.71936 (PMC12373977; doi:10.1002/ece3.71936)
Supplement: Supplementary file 1 — Appendix S1: ece371936‐sup‐0001‐AppendixS1.docx. [file ECE3-15-e71936-s002.docx]

Supplementary Table 1: The abundance and taxonomy information of OTUs assigned to algae in the sampling sites in March.

Supplementary Table 2. The abundance and taxonomy information of OTUs assigned to algae in the sampling sites in July.

Supplementary Table 3. The abundance and taxonomy information of OTUs assigned to algae in the sampling sites in September.

Supplementary Table 4. The abundance and taxonomy information of OTUs assigned to algae in the sampling sites in December.

Supplementary Code S1. A program to extract relative abundance information of major algal phyla.

Supplementary Table 5: The environmental measurements table of the sampling sites in March (3-env).

Supplementary Table 6: The OTUs table of the sampling sites in March (3-OTU).

Supplementary Table 7: The environmental measurements table of the sampling sites in July (7-env).

Supplementary Table 8: The OTUs table of the sampling sites in July (7-OTU).

Supplementary Table 9: The environmental measurements table of the sampling sites in September (9-env).

Supplementary Table 10: The OTUs table of the sampling sites in September (9-OTU).

Supplementary Table 11: The environmental measurements table of the sampling sites in December (12-env).

Supplementary Table 12: The OTUs table of the sampling sites in December (12-OTU).

Supplementary Code S2. Conduct Mantel test analysis using R program to examine the correlation between the environmental matrix (env.txt) and OTU matrix (OTU.txt).

Supplementary Table 13: The table (This file name is referenced in the code as "3-envplsm" ) used for PLS-PM analysis in March.

Supplementary Table 14: The table (This file name is referenced in the code as "7-envplsm") used for PLS-PM analysis in July.

Supplementary Table 15: The table (This file name is referenced in the code as "9-envplsm") used for PLS-PM analysis in September.

Supplementary Table 16: The table (This file name is referenced in the code as "12-envplsm") used for PLS-PM analysis in December.

Supplementary Code S3. Conduct PLS-PM analysis using R program to examine the correlation among latent variables, e.g., seawater properties, nutrients, phytoplankton biomass represented by the Chl-a concentration, alpha diversity, Chlorophyta, and Diatoms.
